# Cerium Oxide Nanoparticles in Lung Acutely Induce Oxidative Stress, Inflammation, and DNA Damage in Various Organs of Mice

**DOI:** 10.1155/2017/9639035

**Published:** 2017-03-14

**Authors:** Abderrahim Nemmar, Priya Yuvaraju, Sumaya Beegam, Mohamed A. Fahim, Badreldin H. Ali

**Affiliations:** ^1^Departments of Physiology, College of Medicine and Health Sciences, United Arab Emirates University, P.O. Box 17666, Al Ain, UAE; ^2^Department of Pharmacology, College of Medicine & Health Sciences, Sultan Qaboos University, P.O. Box 35, Al-Khod, 123 Muscat, Oman

## Abstract

CeO_2_ nanoparticles (CeO_2_ NPs) which are used as a diesel fuel additive are emitted in the particulate phase in the exhaust, posing a health concern. However, limited information exists regarding the* in vivo* acute toxicity of CeO_2_ NPs on multiple organs. Presently, we investigated the acute (24 h) effects of intratracheally instilled CeO_2_ NPs in mice (0.5 mg/kg) on oxidative stress, inflammation, and DNA damage in major organs including lung, heart, liver, kidneys, spleen, and brain. Lipid peroxidation measured by malondialdehyde production was increased in the lungs only, and reactive oxygen species were increased in the lung, heart, kidney, and brain. Superoxide dismutase activity was decreased in the lung, liver, and kidney, whereas glutathione increased in lung but it decreased in the kidney. Total nitric oxide was increased in the lung and spleen but it decreased in the heart. Tumour necrosis factor-*α* increased in all organs studied. Interleukin- (IL-) 6 increased in the lung, heart, liver, kidney, and spleen. IL-1*β* augmented in the lung, heart, kidney, and spleen. Moreover, CeO_2_ NPs induced DNA damage, assessed by COMET assay, in all organs studied. Collectively, these findings indicate that pulmonary exposure to CeO_2_ NPs causes oxidative stress, inflammation, and DNA damage in multiple organs.

## 1. Introduction

Rapid development of nanotechnology led to an immense array of nanomaterials varying in size, shape, charge, chemistry, coating, and solubility. Nanomaterials have different chemical, physical, and biological characteristics compared to larger materials of the same chemical composition. They are now being used widely in biomedical and many industrial applications [1–3]. However, widespread use of nanomaterials may lead to environmental contamination and human exposure by inhalation, dermal and oral routes, raising concerns about their potential toxicity [3]. Amongst these nanomaterials, cerium oxide nanoparticles (CeO_2_ NPs) have a potential for use in industrial, environmental, and pharmaceutical areas. A major environmental usage of CeO_2_ NPs is as a diesel fuel additive to augment fuel efficiency and decrease particulate emissions [4–7]. In fact, CeO_2_ NPs are increasingly used as a fuel-borne catalyst in North America, Europe, and elsewhere [6, 8]. It has been shown that that supplementation of CeO_2_ to diesel reduces fuel consumption by 5%–8% and release of combustion-derived nanoparticles and unburned hydrocarbons by up to 15% [4–7]. Nevertheless, the associated emission of CeO_2_ nanoparticles into the environment may well exert unexpected health effects [8]. Organization for Economic Cooperation and Development has included CeO_2_ NPs in the priority list of the nanomaterials needing urgent assessment [9].

There is disagreement in the published studies about the impact of CeO_2_ NPs on inflammation and oxidative stress. Some studies have reported that CeO_2_ NPs reduce toxicity and inflammation in J774A.1 macrophages [10] and inhibit oxidative stress and nuclear factor-kappaB activation in H9c2 cardiomyocytes exposed to cigarette smoke extract [11]. Also, it has been demonstrated that CeO_2_ NPs protect rodent lungs from hypobaric hypoxia-induced oxidative stress and inflammation in vivo [12]. On the other hand, other studies reported the occurrence of inflammation, oxidative stress, apoptosis, autophagy in vitro [13, 14], and lung inflammation and fibrosis following intratracheal instillation or inhalation of CeO_2_ NPs [15–17]. Moreover, it has been demonstrated that CeO_2_ NPs are able to cross the alveolar capillary barrier and reach extrapulmonary sites following intratracheal (i.t.) instillation or inhalation [17, 18]. However, little is known about the potential pulmonary exposure to CeO_2_ NPs to cause inflammation, oxidative stress, and DNA damage in multiple distant organs. Therefore, the aim of the present study is to comprehensively assess the effect of acute (24 h) i.t. instillation of CeO_2_ NPs in mice on inflammation, oxidative stress, and DNA damage in some vital organs, including the lung, heart, liver, kidney, spleen, and brain.

## 2. Materials and Methods

### 2.1. Particles

CeO_2_ NPs, 10 wt% in water with average diameter at ~20 nm, were obtained from Sigma-Aldrich (St Louis, MO, USA). CeO_2_ NPs samples diluted in saline were used for mouse exposures. To minimize aggregation, particle suspensions were always sonicated for 5 min (Clifton Ultrasonic Bath, Clifton, New Jersey, USA). Particle suspensions were prepared promptly before use and were vortexed to offer well mixed suspension prior to each instillation. The same particles from the same source were characterized and used recently by Ma and coworkers [15, 19].

The endotoxin concentration in the CeO_2_ NPs and saline used was quantified, as described by the manufacturer, by chromogenic Limulus Amebocyte Lysate (Pierce, Rockford, IL) test. The concentrations were lower than the detection limit (0.1 EU/mL) in the saline and CeO2 NPs solutions.

### 2.2. Animals and i.t. Instillation

This project was reviewed and approved by the Institutional Review Board of the United Arab Emirates University, College of Medicine and Health Sciences, and experiments were performed in accordance with protocols approved by the Institutional Animal Care and Research Advisory Committee.

Both male and female BALB/C mice (body weight: 23 ± 2 g) (Taconic Farms Inc., Germantown, NY, USA) were housed in light (12 h light : 12 h dark cycle) and temperature-controlled (22 ± 1°C) rooms. They had free access to commercial laboratory chow and were provided tap water ad libitum.

**Figure 1:**
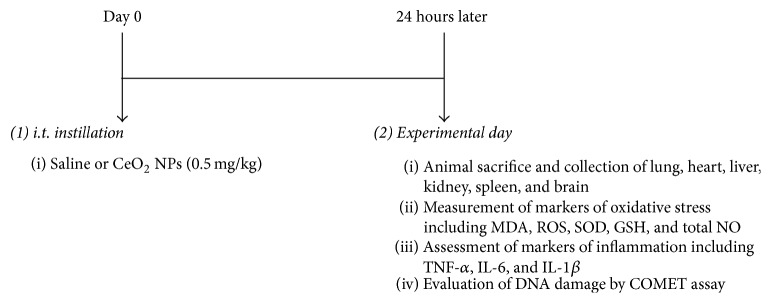
Treatments and endpoints assessed including markers of oxidative and nitrosative stress [malondialdehyde (MDA), reactive oxygen species (ROS), superoxide dismutase (SOD), glutathione (GSH), and total nitric oxide (NO)], inflammation [tumor necrosis factor-*α* (TNF-*α*), interleukin-6 (IL-6), and IL-1*β*], and DNA damage by COMET assay, 24 h after intratracheal instillation of saline (control) or 0.5 mg/kg cerium oxide nanoparticles (CeO_2_ NPs) in mice.

Mice were anesthetized with sodium pentobarbital (60 mg/kg, i.p.) and placed supine with extended neck on an angled board. A Becton Dickinson 24 Gauge cannula was inserted via the mouth into the trachea. The CeO_2_ NPs suspensions (0.5 mg/kg) or saline-only were instilled intratracheally (i.t.) (100 *μ*L) via a sterile syringe and followed by an air bolus of 100 *μ*L, and 24 h later several endpoints were measured (Figure 1). The experiments were repeated three times.

### 2.3. Assessment of Tissue Lipid Peroxidation (LPO) Measured by Malondialdehyde (MDA) Production, Reactive Oxygen Species (ROS), Superoxide Dismutase (SOD), Glutathione (GSH), and Total Nitric Oxide (NO)

Twenty-four hours after the i.t. administration of saline or CeO_2_ NPs, the mice were sacrificed by an overdose of sodium pentobarbital. Immediately after that, lungs, heart, liver, kidney, spleen, and brain were quickly collected and rinsed with ice-cold PBS (pH 7.4) before homogenization in 0.1 M phosphate buffer, pH 7.4, containing 0.15 M KCl, 0.1 mM EDTA, 1 mM DTT, and 0.1 mM phenylmethylsulfonyl fluoride at 4°C. The homogenates were centrifuged at 14,000 rpm for 20 min at 4°C, and protein was measured as reported before [20–22].

LPO measured by MDA production in homogenates obtained from all organs studied was determined colorimetrically following its controlled reaction with thiobarbituric acid using TBARS kit purchased from Cayman Chemical Company (Ann Arbor, MI, USA).

ROS were measured in the homogenates from all organs studied using 2′,7′-dichlorofluorescein diacetate (DCFDA, Molecular Probes, Eugene, OR, USA) as a fluorescent probe as described before [20, 21, 23]. The results were normalized as ROS produced per mg of protein.

SOD activity was measured as the conversion of nitroblue tetrazolium (NBT) to NBT-diformazan according to the vendor's protocol (R&D System, MN, USA). The extent of reduction in the appearance of NBT-formazan was used as a measure of SOD activity present in each organ [20–22].

GSH concentration was measured using a commercially available kit (Sigma-Aldrich Fine Chemicals, St Louis, MO, USA).

The determination of nitric oxide (NO) was performed with a total NO assay kit from R&D systems (Minneapolis, MN, USA) which measures the more stable NO metabolites NO_2_^−^ and NO_3_^−^ [24, 25].

### 2.4. Measurement of Interleukin-6 (IL-6), IL-1*β*, and Tumor Necrosis Factor-*α* (TNF-*α*) in Tissues

In separate experiments, animals were sacrificed by an overdose of sodium pentobarbital, and their lung, heart, liver, kidney, spleen, and whole brain were quickly collected and rinsed with ice-cold PBS (pH 7.4) before homogenization in 50 mM Tris buffer, pH 7.4, containing 400 mM NaCl and 0.5% Triton X-100 at 4°C [26]. The homogenates were centrifuged at 14,000 rpm for 15 min at 4°C to remove cellular debris, and the supernatants were used for further analysis. Protein content in each organ was measured by Bradford's method, as described before [20, 21]. The concentrations of IL-6, IL-1*β*, and TNF *α* in the tissues were determined using ELISA kits (Duo Set, R&D systems, Minneapolis, MN, USA).

### 2.5. DNA Damage Assessment by COMET Assay

Immediately after sacrifice, the lung, heart, liver, kidney, spleen, and brain were removed from each animal. Single-cell suspensions of the different lungs, hearts, livers, kidneys, spleens, and brains were obtained and analyzed according to the method described in our previous publications [27–30].

Each collected organ was washed in a chilled medium (RPMI 1640, 15% DMSO, and 1.8% (w/v) NaCl). The lung, heart, liver, kidney, spleen, and brain tissues were put in 1.5 mL medium and cut finely into pieces in a Petri dish. The slices were allowed to deposit and the supernatant was collected in a 15 mL tube. The collected cell suspension was centrifuged at 1000 rpm for 5 min at 4°C. The supernatant was removed and the pellets were suspended in 0.5 mL of the medium. The cell suspensions were mixed with low melting point agarose solution (0.65%) and spread onto agarose (1.5%)-precoated microscope slides. For each group, five slides were prepared and incubated in ice-cold lysis buffer (2.5 M NaCl, 10 mM Tris, 100 mM EDTA, 1% Triton X-100, and 10% DMSO) at 4°C for at least one hour to remove the cell membranes. Following incubation, slides were placed in a horizontal electrophoresis unit and incubated in electrophoresis buffer (0.2 M EDTA, 5 M NaCl, pH 10) for 20 min for DNA unwinding and the expression of alkali labile sites. Then, electrophoresis was conducted for 20 min at 25 V and 300 mA. After that, the slides were neutralized with Tris buffer (0.4 M Trizma base, pH 7.5) for 5 min and washed with methanol. Then the slides were stained with propidium iodide, as previously described [28, 31]. All these steps were performed in darkness to prevent additional DNA damage. The slides were mounted on a fluorescent microscope and cell scoring was performed. The measurement of length of the DNA migration (i.e., diameter of the nucleus plus migrated DNA) was calculated using the image analysis Axiovision 3.1 software (Carl Zeiss, Canada) [28, 32].

### 2.6. Statistics

All statistical analyses were performed with GraphPad Prism Software version 5. Data were analyzed using the unpaired* t*-test for differences between groups. All the data in figures are reported as mean ± SEM. *P* values < 0.05 are considered significant.

## 3. Results

### 3.1. Effect of CeO_2_ NPs on the Release of MDA, ROS, SOD, GSH, and Total NO in the Lung, Heart, Liver, Kidney, Spleen, and Brain

**Figure 2:**
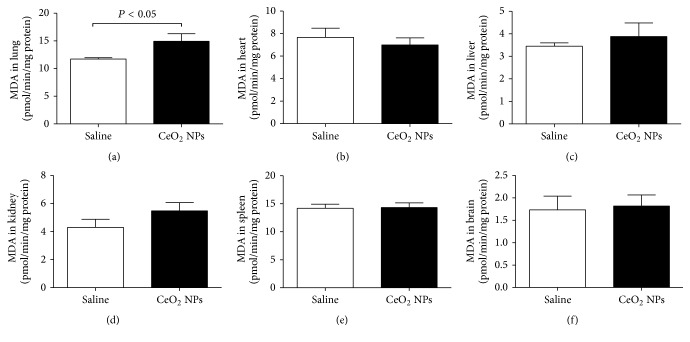
Lipid peroxidation measured by malondialdehyde (MDA) production in the lung (a), heart (b), liver (c), kidney (d), spleen (e), and brain (f), 24 h after intratracheal instillation of saline (control) or 0.5 mg/kg cerium oxide nanoparticles (CeO_2_ NPs) in mice. Data are mean ± SEM (*n* = 8 in each group).

**Figure 3:**
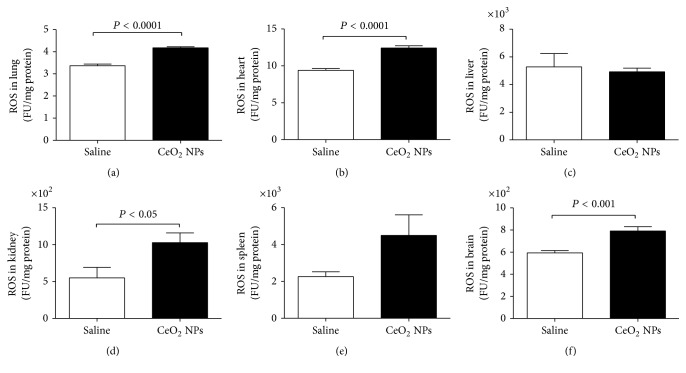
Reactive oxygen species (ROS) in the lung (a), heart (b), liver (c), kidney (d), spleen (e), and brain (f), 24 h after intratracheal instillation of saline (control) or 0.5 mg/kg cerium oxide nanoparticles (CeO_2_ NPs) in mice. Data are mean ± SEM (*n* = 7-8 in each group).

**Figure 4:**
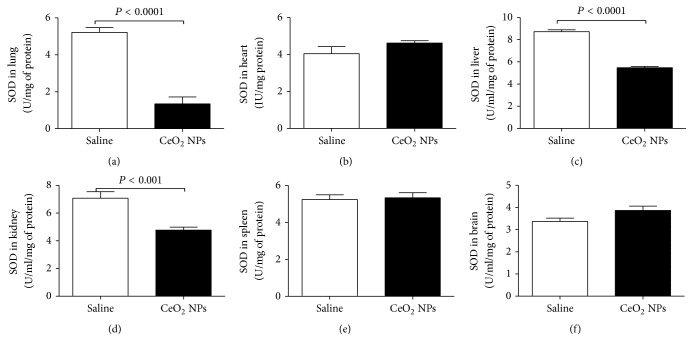
Superoxide dismutase (SOD) activities in the lung (a), heart (b), liver (c), kidney (d), spleen (e), and brain (f), 24 h after intratracheal instillation of saline (control) or 0.5 mg/kg cerium oxide nanoparticles (CeO_2_ NPs) in mice. Data are mean ± SEM (*n* = 8 in each group).

**Figure 5:**
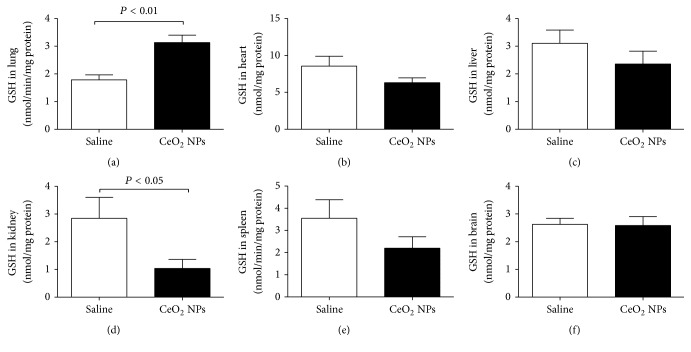
Glutathione (GSH) concentrations in the lung (a), heart (b), liver (c), kidney (d), spleen (e), and brain (f), 24 h after intratracheal instillation of saline (control) or 0.5 mg/kg cerium oxide nanoparticles (CeO_2_ NPs) in mice. Data are mean ± SEM (*n* = 8 in each group).

**Figure 6:**
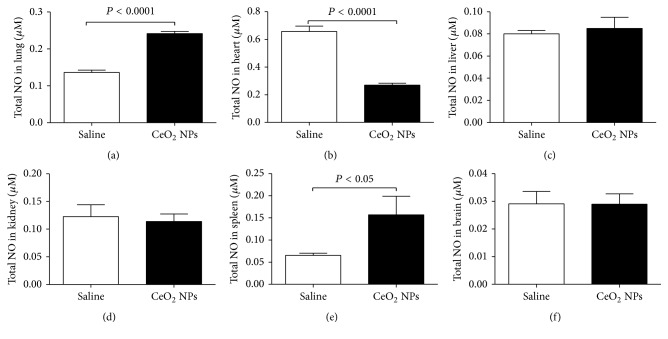
Total nitric oxide (NO) in the lung (a), heart (b), liver (c), kidney (d), spleen (e), and brain (f), 24 h after intratracheal instillation of saline (control) or 0.5 mg/kg cerium oxide nanoparticles (CeO_2_ NPs) in mice. Data are mean ± SEM (*n* = 6–8 in each group).

We measured ROS and MDA in several organs. The latter is a well-known mechanism of cellular injury and is utilized as a marker of oxidative stress in cells and tissues [33]. In addition, we quantified the concentration of GSH, a free radical scavenger, and the activity of a key antioxidant enzyme, namely, SOD, which is effective in dismutating O_2_^−∙^ to H_2_O_2_ [33]. The total NO was also assessed as a marker of nitrosative stress. The effects of CeO_2_ NPs on the aforementioned markers of oxidative and nitrosative stress in the lung, heart, liver, kidney, spleen, and brain are illustrated in Figures 2–6. The concentration of MDA was significantly increased in the lung (*P* < 0.05) but it was not affected in the heart, liver, kidney, spleen, and brain (Figure 2). ROS levels were significantly increased in the lung (*P* < 0.0001), heart (*P* < 0.0001), kidney (*P* < 0.05), and brain (*P* < 0.001) (Figure 3). The activity of the antioxidant SOD was significantly reduced in the lung (*P* < 0.0001), liver (*P* < 0.0001), and kidney (*P* < 0.001) (Figure 4). The concentration of GSH was significantly increased in the lung (*P* < 0.01) but it was significantly decreased in the kidney (*P* < 0.05) (Figure 5). The total NO was augmented in the lung (*P* < 0.0001) and spleen (*P* < 0.05) but it was decreased in the heart (*P* < 0.0001) (Figure 6).

### 3.2. Effect of CeO_2_ NPs on the Levels of TNF-*α*, IL-6, and IL-1*β* in the Lung, Heart, Liver, Kidney, Spleen, and Brain

**Figure 7:**
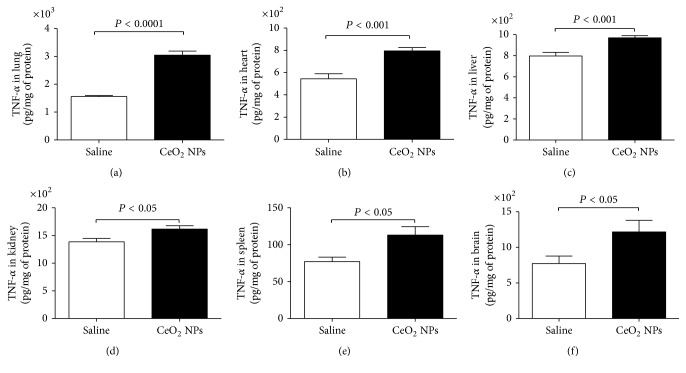
Tumor necrosis factor-*α* (TNF-*α*) concentrations in the lung (a), heart (b), liver (c), kidney (d), spleen (e), and brain (f), 24 h after intratracheal instillation of saline (control) or 0.5 mg/kg cerium oxide nanoparticles (CeO_2_ NPs) in mice. Data are mean ± SEM (*n* = 6–8 in each group).

**Figure 8:**
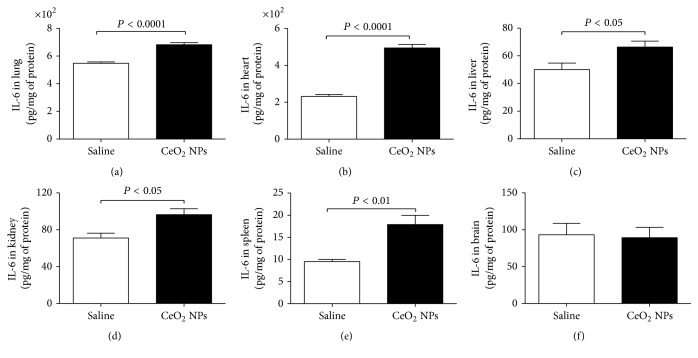
Interleukin-6 (IL-6) concentrations in the lung (a), heart (b), liver (c), kidney (d), spleen (e), and brain (f), 24 h after intratracheal instillation of saline (control) or 0.5 mg/kg cerium oxide nanoparticles (CeO_2_ NPs) in mice. Data are mean ± SEM (*n* = 8 in each group).

**Figure 9:**
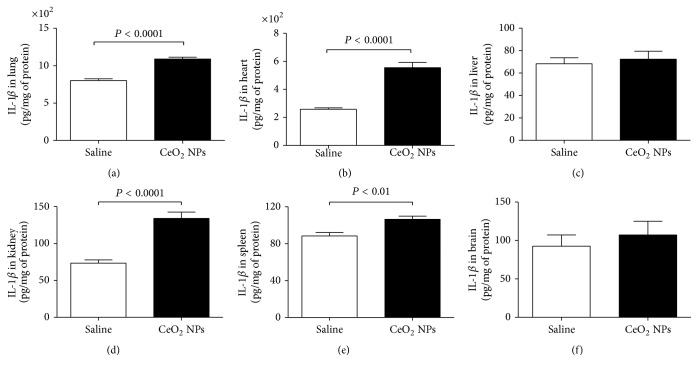
Interleukin-1*β* (IL-1*β*) concentrations in the lung (a), heart (b), liver (c), kidney (d), spleen (e), and brain (f), 24 h after intratracheal instillation of saline (control) or 0.5 mg/kg cerium oxide nanoparticles (CeO_2_ NPs) in mice. Data are mean ± SEM (*n* = 8 in each group).

TNF-*α*, IL-6, and IL-1*β* are proinflammatory cytokines which were reported to be upregulated in the lungs of mice and rats exposed to nanoparticles [34]. The impact of CeO_2_ NPs on the above-mentioned proinflammatory cytokines in the lung, heart, liver, kidney, spleen, and brain is illustrated in Figures 7–9.

Compared with the control group, the concentration of TNF-*α* was significantly increased by i.t. administration of CeO_2_ NPs in all studied organs, that is, lung (*P* < 0.0001), heart (*P* < 0.001), liver (*P* < 0.001), kidney (*P* < 0.05), spleen (*P* < 0.05), and brain (*P* < 0.05) (Figure 7).

The concentration of IL-6 was significantly increased by CeO_2_ NPs exposure in the lung (*P* < 0.0001), heart (*P* < 0.0001), liver (*P* < 0.05), kidney (*P* < 0.05), and spleen (*P* < 0.01) (Figure 8).

Compared with saline-instilled group, IL-1*β* concentration was significantly increased by the pulmonary exposure to CeO_2_ NPs in the lung (*P* < 0.0001), heart (*P* < 0.0001), kidney (*P* < 0.0001), and spleen (*P* < 0.01) (Figure 9).

### 3.3. Effect of CeO_2_ NPs on the DNA Damage in the Lung, Heart, Liver, Kidney, Spleen, and Brain

**Figure 10:**
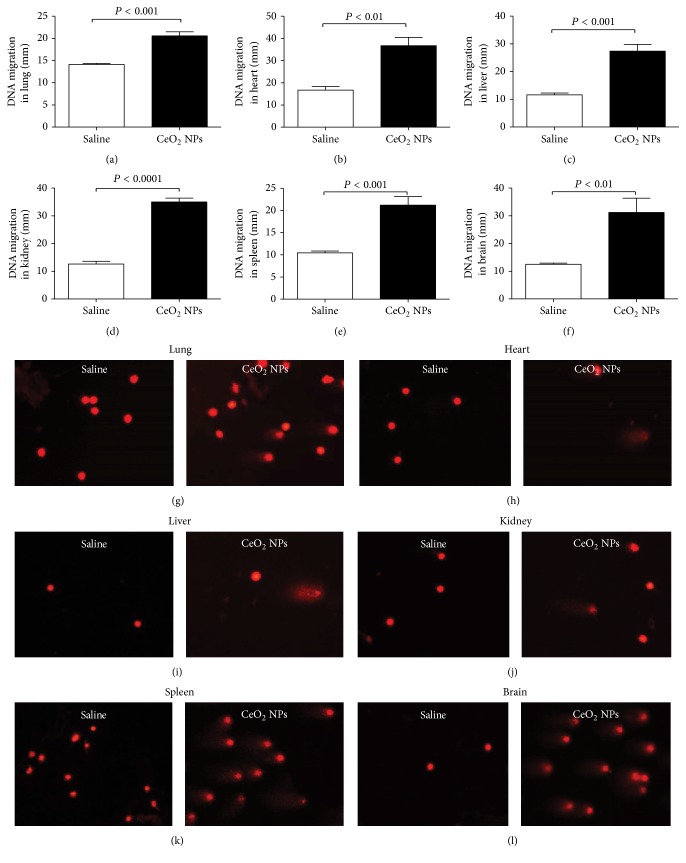
DNA migration in the lung (a), heart (b), liver (c), kidney (d), spleen (e), and brain (f), 24 h after intratracheal instillation of saline (control) or 0.5 mg/kg cerium oxide nanoparticles (CeO_2_ NPs) in mice. Data are mean ± SEM (*n* = 5 in each group). Images illustrating the quantification of the DNA migration by the COMET assay under alkaline conditions in lung (g), heart (h), liver (i), kidney (j), spleen (k), and brain (l) tissues.

To evaluate the DNA damage in cells, we applied the single-cell gel electrophoresis (COMET assay). In electrophoresis, under alkaline conditions, cells exhibiting DNA damage show augmented migration of DNA emerging from strand breaks. Damaged DNA migrates more under the influence of the electric field, and the nucleoids look like “comets,” with the head and a bright fluorescent tail. The magnitude of the DNA migration is directly associated with the degree of DNA impairment [28, 31, 35].  Figure 10 shows the effect of CeO_2_ NPs on DNA damage in the studied organs assessed by COMET assay. Compared with saline-treated group, i.t. administration of CeO_2_ NPs induced a significant DNA migration in the lung (*P* < 0.001, Figure 10(a)), heart (*P* < 0.01, Figure 10(b)), liver (*P* < 0.001, Figure 10(c)), kidney (*P* < 0.0001, Figure 10(d)), spleen (*P* < 0.001, Figure 10(e)), and brain (*P* < 0.01, Figure 10(f)).

## 4. Discussion

In this study, we have shown that acute (24 h) i.t. administration of CeO_2_ NPs caused oxidative stress, inflammation, and DNA damage in several major organs, including lung, heart, liver, kidney, spleen, and brain.

Nanotechnology has demonstrated merit in advancing quality of everyday life and has resulted in the manufacturing of a wide range of nanomaterials for various purposes, including medical, industrial, and consumer product uses [3]. Nevertheless, there is a deficiency of satisfactory data about the impact of these nanomaterials on human health and the environment [3]. The CeO_2_ NPs are being increasingly used in industry as oxidation catalyst, gas sensor, polishing materials, and UV absorber. In the petroleum refining industry, CeO_2_ NPs are used as additives to promote combustion of diesel fuels, automotive exhaust cleaning, and electrolytes in solid oxide fuel cells [4–7]. It has been shown that the use of cerium compounds as diesel fuel catalyst results in the production in the CeO_2_ NPs in the exhaust [8], warranting toxicological assessment of these nanoparticles on the lung and secondary organs.

In the present study, we assessed the acute (24 h) pulmonary and extrapulmonary effects of CeO_2_ NPs following i.t. instillation. This time point is similar to that used to assess the cardiovascular effects of i.t. administered CeO_2_ NPs [36]. This is relevant to clinical and experimental studies which have previously shown the occurrence of cardiovascular dysfunction within 24 h of exposure to increased levels of particulate air pollution [37–39]. In the current study, we used i.t. administration of nanoparticles because it provides more accurate dosing, given that mice are nose breathers that filter most inhaled particles [40, 41]. The dose of CeO_2_ NPs used in the present study has been selected from previous studies involving animal models of i.t. or oropharyngeal instillation of CeO_2_ NPs and which assessed the impact of these nanoparticles on lung inflammation and alveolar macrophage functional change in rats and vascular reactivity and ischemia-reperfusion injury in mice [15, 36].

Several studies have reported the occurrence of lung inflammation and oxidative stress and nanoparticle translocation and accumulation in secondary organs, such as the liver, following i.t. instillation or inhalation CeO_2_ NPs [15–18, 42]. However, as far as we are aware, no study has investigated systematically the oxidative stress (LPO, ROS, SOD, GSH, and total NO), inflammation (TNF-*α*, IL-6, and IL-1*β*), and DNA damage in the lung and important secondary organs including heart, liver, kidney, spleen, and brain.

Oxidative stress is a consequence of imbalance between the levels of antioxidants and ROS. To maintain redox equilibrium, cells are able to balance the production of oxidants and antioxidants. Oxidative stress happens when this balance is affected by excessive production of ROS and/or depletion of antioxidant protections [43]. The available data regarding the effects of CeO_2_ NPs on oxidative stress is contradictory. While studies reported that CeO_2_ NPs have antioxidant properties and protect against oxidative stress induced by cigarette smoke and X-ray radiation, as well as, in animal models, ischemia/reperfusion, stroke, and neurodegeneration, other studies have, however, reported that these nanoparticles cause oxidative stress both in vivo following pulmonary exposure and in vitro in human monocytes [44]. The reason behind these discrepancies could be related to the preparation methods of CeO_2_ NPs, the pH of the biological milieu in which the nanoparticles are tested, the particle size, the cell types, and the route of exposure [44]. Our data show that, as a consequence of the route of exposure to CeO_2_ NPs, that is, through the lung, all the markers of oxidative stress assessed were affected in the lung tissue. In fact, MDA and ROS were significantly increased in lung tissue, indicating the occurrence of oxidative stress. The antioxidant SOD was significantly decreased by CeO_2_ NPs, whereas that of GSH was increased. The decrease of SOD activity indicates that it has been consumed as a result of oxidative stress [22, 45, 46]. The increase of GSH in the lung suggests that the development of oxidative stress is followed by an adaptive reaction that balances the potentially damaging activity of ROS by antioxidant defence mechanisms [28, 47]. It has been recently reported that intraperitoneal administration of gold nanoparticles in rats induces an increase of GSH and a decrease of SOD in lung [48]. ROS was significantly increased in the heart, kidney, and brain. Also, our data show that SOD was decreased in the liver and kidney, and GSH was decreased in the kidney. A previous report has demonstrated a decrease of SOD activity in the kidney and liver following exposure to gold nanoparticles in rats [48]. Moreover, GSH concentration was also found to decrease in the kidney of rats after oral administration of silver nanoparticles [49]. Along with that, we observed a significant increase in the total NO in the lung and spleen but a decrease in the heart. An increase NO production by pulmonary cells following silica nanoparticles exposure has been previously described [50]. However, a decrease of NO in the heart has been reported following i.t. instillation of silica nanoparticles in rats [51].

In the present study, besides measuring markers of oxidative stress, we also provide evidence that pulmonary exposure to CeO_2_ NPs induced a significant augmentation in the concentrations of TNF-*α* in all studied organs; IL-6 in the lung, heart, liver, kidney, and spleen; and IL-1*β* in the lung, heart, kidney, and spleen. Even though the release of markers of inflammation varied between the investigated organs, at least one or more than one marker of inflammation augmented in each studied organ, showing the occurrence of inflammation after i.t. instillation of CeO_2_ NPs. Inflammation and oxidative stress are closely related pathophysiological processes [52, 53]. We presently found a significant increase of TNF-*α*, IL-6, and IL-1*β* in the lung, heart, and kidney, and this has coincided with the increase of ROS in the aforementioned organs. The latter should have supposedly resulted in increase of LPO in these organs. Yet, we found that the marker of LPO measured as MDA had only increased in the lung but not in the heart and kidney. This finding cannot be readily explained but it is worth mentioning that, in the present study, we assessed the pulmonary and extrapulmonary effects of i.t. instilled CeO_2_ NPs. Since the employed nanoparticles were first deposited directly in the lung, the manifestations of the biochemical insult on this organ were higher than in the distant organs, possibly due to the higher amount of nanoparticles deposited directly in the lung tissue, compared to other organs. That is why the effects observed in this organ were all consistent and significant. Nevertheless, the effects on distant organs were variable for the markers of inflammation and oxidative stress measured. The latter could be explained by the complexity of the mechanisms of action of pulmonary deposited nanoparticles on extrapulmonary organs which can result from translocation of small amount of nanoparticles across the alveolar capillary barrier and their accumulation in secondary organs [34]. Another possible mechanism of action is related to pulmonary inflammation caused by these nanoparticles in the lung which elicits systemic release of cytokines that may influence distant organs to variable degrees [34]. In the lung, we found an increase of both ROS and MDA. Nevertheless, in other organs such as the heart and kidney, while ROS was increased, MDA was not significantly increased. While we cannot exclude the contribution of ROS, the observed effect could be related, at least partly, to the direct impact of CeO_2_ NPs on cell membranes in the lung and that the small proportion of these nanoparticles which have presumably translocated and reached extrapulmonary organs were not sufficient to trigger lipid peroxidation. Oxidative stress induced by engineered nanoparticles can be related to acellular factors such as particle surface, dose, size and composition, and direct nanoparticle-cell interactions [54]. Another possible explanation is that the lack of increase of MDA in heart and kidney does not completely exclude a possible increase of other markers of lipid peroxidation such as conjugated dienes, ethane and pentane gases, isoprostanes, and 4-hydroxynonenal. Additional work, using multiple indices of lipid oxidation and oxidative and nitrosative stress, is warranted to clarify this matter.

The evaluation of markers of inflammation and oxidative stress in multiple organs after pulmonary exposure to CeO_2_ NPs has not been documented before. However, it has been shown that exposure to CeO_2_ NPs by instillation or inhalation is associated with increased liver ceria levels, reductions in liver weight, evidence of liver damage, and increase in TNF-*α* concentration in plasma [17, 55].

Since we observed the occurrence of inflammation and oxidative stress, following lung exposure to CeO_2_ NPs, we wanted to assess whether and to what extent CeO_2_ NPs can induce DNA damage in the studied organs using gel electrophoresis of a single cell (COMET assay). Our data show the occurrence of DNA damage in all the studied organs following i.t. instillation of CeO_2_ NPs. The DNA damage, inflammation, and oxidative stress observed in the brain could at least partly result from the translocation [18, 56] and, hence, the direct effect of these nanoparticles on the brain. Although the mechanism related to nanoparticle translocation into the brain following pulmonary exposure is not fully understood, it has been shown that nanoparticles can reach the central nervous system using sensory nerves present in the upper respiratory tract and tracheobronchial region and some in the alveolar region [57]. The latter pathway bypasses the very firm blood brain barrier [57]. This translocation has been shown to be affected by the size and surface chemistry of the nanoparticles [57]. Since DNA damage was consistently observed in all the studied organs, it is possible that DNA damage might be an early step of CeO_2_ NPs-induced toxicity, which will eventually lead to oxidative stress and inflammation. It is also likely that the DNA damage results from the inflammation and/or oxidative stress which took place in the different organs studied. It has been reported that oral administration of high dose (1000 mg/kg bw) of CeO_2_ NPs induces significant DNA damage in peripheral blood leukocytes and liver cells, micronucleus formation in bone marrow and blood cells, and total cytogenetic changes in bone marrow [58]. Moreover, it has been shown that CeO_2_ NPs induce oxidative stress and genotoxicity in human skin melanoma cells and human dermal fibroblasts [59, 60]. A more recent study has shown that low and more relevant concentration of CeO_2_ NPs (0.01 mg/L) adversely affected in vitro fertilization in mice and caused DNA damage in mouse spermatozoa and oocytes [61]. The observed DNA damage was explained by the direct impact of CeO_2_ NPs and/or mechanical impact on the disruption of gamete interactions. In fact, the same research group has demonstrated earlier the accumulation of CeO_2_ NPs along oocyte zona pellucida [62]. Finally, it has also been suggested that CeO_2_ NPs could negatively impact fertilization by causing oxidative stress [61].

We conclude that acute pulmonary exposure to CeO_2_ NPs causes oxidative stress, inflammation, and DNA damage in multiple major organs, including the lung, heart, liver, kidney, spleen, and brain. Additional studies are warranted to investigate the time and dose effects and the mechanisms underlying the observed effects.
